# Utility of T-Cell Interferon-γ Release Assays for Etiological Diagnosis of Classic Fever of Unknown Origin in a High Tuberculosis Endemic Area — a pilot prospective cohort

**DOI:** 10.1371/journal.pone.0146879

**Published:** 2016-01-19

**Authors:** Xiaochun Shi, Lifan Zhang, Yueqiu Zhang, Baotong Zhou, Xiaoqing Liu

**Affiliations:** 1 Department of Infectious Diseases, Peking Union Medical College Hospital, Chinese Academy of Medical Sciences & Peking Union Medical College, Beijing, China; 2 Clinical Epidemiology Unit, Peking Union Medical College, International Clinical Epidemiology Network, Beijing, China; Chinese Academy of Medical Sciences, CHINA

## Abstract

**Background:**

Tuberculosis (TB), especially extrapulmonary TB is still the leading cause of fever of unknown origin (FUO) in China. However, diagnosis of TB still remains a challenge. The aim of this study was to evaluate the diagnostic value of T-SPOT.*TB* for etiological diagnosis of classic FUO in adult patients in a high TB endemic area.

**Methods:**

We prospectively enrolled patients presenting with classic FUO in a tertiary referral hospital in Beijing, China, to investigate the diagnostic sensitivity, specificity, predictive values and likelihood ratio of T-SPOT.*TB*. Clinical assessment and T-SPOT.*TB* were performed. Test results were compared with the final confirmed clinical diagnosis.

**Results:**

387 hospitalized patients (male n = 194, female n = 193; median age 46 (range 29–59) yrs) with classic FUO were prospectively enrolled into this study. These FUOs were caused by infection (n = 158, 40.8%), connective tissue disease (n = 82, 21.2%), malignancy (n = 41, 10.6%) and miscellaneous other causes (n = 31, 8.0%), and no cause was determined in 75 (19.4%) patients. 68 cases were diagnosed as active TB eventually. The sensitivity of T-SPOT.*TB* for the diagnosis of active TB was 70.6% (95%CI 58.9–80.1%), while specificity was 84.4% (95%CI 79.4–88.4%), positive predictive value was 55.8% (95%CI 45.3–65.8%), negative predictive value was 91.2% (95%CI 86.7–94.2%). Among these 68 active TB patients, 12 cases were culture or histology confirmed (11 cases with positive T-SPOT.*TB*, sensitivity was 91.7%) and 56 cases were clinically diagnosed (37 cases with positive T-SPOT.*TB*, sensitivity was 66.1%); 14 cases were pulmonary TB (13 cases with positive T-SPOT.*TB*, sensitivity was 92.9%) and 54 cases were extrapulmonary TB (35 cases with positive T-SPOT.*TB*, sensitivity was 64.8%).

**Conclusions:**

For patients presenting with classic FUO in this TB endemic setting, T-SPOT.*TB* appears valuable for excluding active TB, with a high negative predictive value.

## Introduction

In 1961, fever of unknown origin (FUO) was defined by Petersdorf and Beeson as an illness of more than 3 weeks’ duration, fever greater than 38.3°C (101°F) on several occasions, and diagnosis uncertain after 1 week of observation in hospital [1]. In 1991, Durack et al suggested that FUO can be subclassified into four different types: classic FUO, nosocomial FUO, immune-deficient FUO and HIV-related FUO, as helpful for determination of probable causes of FUO [2]. The common causes of classic FUO are infections, connective tissue diseases (CTD), neoplasms and miscellaneous diseases. Unfortunately, some cases end without a diagnosis despite exhaustive workup. According to Horowitz’s report in 2013, there were remarkable changes of major causes of FUO during the past 60 years; the proportions of infection and neoplasm are descending, but, despite improved serological and imaging technologies, more FUOs have actually eluded diagnosis in recent years [3]. Different from foreign reports, infection are still the leading etiology of FUO in China [4–6]. Among infectious diseases, tuberculosis (TB), especially extrapulmonary TB, was the leading cause of FUO in China [4–6]. So, it’s important for us to increase the diagnostic efficiency and rapidity of TB among these FUO patients.

TB can happen in people of all ages with various clinical manifestations. Identification of the specific mycobacterium by culture or microscopy in clinical samples has been considered the gold standard for the diagnosis of TB. However, there are many disadvantages of conventional laboratory tests including long lag time, poor sensitivity and invasive procedures, rendering these diagnostic methods unsuitable for routine practice. Therefore, many patients are diagnosed according to their clinical presentation and their response to anti-TB therapy and so need a long time to reach a diagnosis. The median time interval from onset of fever to diagnosis is 19 weeks according to our previous report on TB first presenting as FUO [7].

Interferon-gamma release assays (IGRA), which detects interferon-γ response to *Mycobacterium tuberculosis* (MTB) specific antigens encoded in the RD1 region, have been developed as a sensitive, specific and rapid immunodiagnostic test for TB infection. T-SPOT.*TB* is an enzyme-linked immunospot assay performed on separated and counted peripheral blood mononuclear cells (PBMCs); it uses MTB specific antigens including early secreted antigenic target 6 (ESAT-6) and culture filtrate protein (CFP-10) peptides, and the result is reported as number of interferon-gamma producing T cells (spot-forming cells).

The aim of the present study is to conduct a prospective cohort study in a high TB endemic area to evaluate the diagnostic value of T-SPOT.*TB* for diagnosing active tuberculosis (ATB) among classic FUO adult patients.

## Methods

### Ethics statement

This study was approved by the Ethics Committee of Peking Union Medical College Hospital. Written informed consent was obtained from all patients enrolled in this study.

### Patients and study procedures

This prospective study was conducted in Peking Union Medical College Hospital in China from September 2010 to August 2013. All adult patients (≥16 years old) admitted to the infectious disease ward with classic FUO were included into the study. For the purposes of the study, FUO was defined by the classical criteria: (1) temperature of >38.3°C (>101°F) on several occasions; (2) a duration of fever of >3 weeks; and (3) failure to reach a diagnosis despite three outpatient visits or three days in the hospital without elucidation of a cause. Exclusion criteria were as follows: (1) nosocomial FUO; (2) patients known to have HIV infection; (3) patients with known malignancy; and (4) patients on steroids or immunosuppressants.

Patients were evaluated with routine diagnostic work-up. Clinical information was extracted from patients’ medical records by researchers blinded to the T-SPOT.*TB* results who also tracked patients’ final diagnosis. ATB was defined by the presence of either of the following criteria: (1) clinical specimens were positive for MTB on culture or acid-fast stain; (2) histological biopsy showed epithelioid granuloma with or without caseous necrosis and no other causes other than TB; (3) patients showed successful responses to anti-TB therapy with consistent with clinical manifestation of ATB and exclusion of alternative diagnosis.

A 4ml peripheral blood sample was collected from each patient. Specific T cell responses to RD1 encoded antigens were detected by T-SPOT.*TB* (Oxford Immunotec, Abingdon, UK) that was performed within 6 hours from sample collection by laboratory personnel blinded to patients’ clinical data. T SPOT.*TB* utilized AIM-V (GIBCO^™^ AIM V Medium liquid, Invitrogen, USA.) as negative control, PHA as positive control, and ESAT-6 and CFP-10 as specific antigens, respectively. PBMCs were obtained from each subject for T-SPOT.*TB* and were plated (2.5×10^5^ per well) on a plate precoated with antibody against interferon γ. Plates were incubated for 16–18h at 37°C in 5% carbon dioxide. After incubation, wells were developed with a conjugate against the antibody used and an enzyme substrate. Spot-forming cells (SFCs) were counted with an automated ELISPOT reader (AID-ispot, Strassberg, Germany), each SFC represented an antigen-specific T cell secreting interferon γ. The response was considered positive when the antigen well contained 6 or more spots and had twice the number of spots than the negative control well. The background number of spots in negative control well for PBMC should be less than 10 spots.

### Statistical analysis

Sensitivity, specificity, positive predictive value (PPV), negative predictive value (NPV), positive likelihood ratio (PLR), and negative likelihood ratio (NLR) were calculated to evaluate diagnostic performance for the T-SPOT.*TB*. The difference in means was assessed using Students’ t-tests or rank sum test. The Pearson’s Chi-square test was used to compare the positive proportions. Ninety-five percent confidence intervals (95%CI) were estimated according to the binomial distribution. Significance was inferred for P<0.05. Analyses were performed using statistical software SPSS 16.0.

## Results

387 hospitalized patients with classic FUO were prospectively enrolled into this study. Demographic characteristics are shown in Table 1. FUOs were determined to be caused by infection (n = 158, 40.8%), connective tissue disease (n = 82, 21.2%), malignancy (n = 41, 10.6%) and miscellaneous other causes (n = 31, 8.0%); and no cause was determined in 75 (19.4%) patients. ATB was diagnosed in 68 (17.6%) cases and ATB was excluded in 244 (63.0%) cases. Age and duration of fever were not significant different between these groups. Among patients with ATB, more patients were male and had a previous history of TB.

**Table 1:** Demographic and characteristics of the study population.

	Total	Active tuberculosis	Active tuberculosis excluded	Cause undertermined	P
	n = 387	n = 68	n = 244	n = 75	
Age(years),(median IQR)	46(29–59)	49(36–62)	44.5(26–59)	48(35–60)	0.097
Gender					0.014
Male(%)	194(50.1%)	45(66.2%)	115(47.1%)	34(45.3%)	
Female(%)	193(49.9%)	23(33.8%)	129(52.9%)	41(54.7%)	
Evidences of previous TB(%)	47(12.1%)	20(29.4%)	19(7.8%)	8(10.7%)	<0.001
Contact history of pulmonary TB(%)	2(0.5%)	1(1.5%)	1(0.4%)	0(0)	/
Duration of fever^a^(days),(median IQR)	/	93.5(54.25–165.25)	80.5 (48–165.50)	/	0.386

^a^Duration of fever: period from onset to diagnosis of the disease.

There was no inconclusive T-SPOT.*TB* result on PBMC. Diagnostic parameters of T-SPOT.*TB* among patients with FUO were shown in Table 2. The sensitivity of T-SPOT.*TB* for diagnosis of (clinically defined) ATB was 70.6%, while specificity was 84.4%, PPV was 55.8%, NPV was 91.2%. Among these 68 ATB patients, 12 cases were culture or histology confirmed TB (11 cases with positive T-SPOT.*TB*, sensitivity was 91.7%) and 56 cases were clinically diagnosed TB (37 cases with positive T-SPOT.*TB*, sensitivity was 66.1%); 14 cases were pulmonary TB (13 cases with positive T-SPOT.*TB*, sensitivity was 92.9%) and 54 cases were extra pulmonary TB. In addition, we conduct additional sensitivity analysis. When all patients without definitive diagnosis were considered as patients with exclusion of ATB, the sensitivity of T-SPOT.TB for the diagnosis of ATB was 70.6% (95%CI 58.3–81.0%), while specificity was 80.9% (95%CI 76.1–85.1%), positive predictive value was 44.0% (95%CI 34.5–53.9%), negative predictive value was 92.8% (95%CI 89.1–95.6%), which results are consistent with the primary analysis.

**Table 2:** Diagnostic parameters of T-SPOT.*TB* among patients with classic FUO.

	Total	T.SPOT-TB(+)	T.SPOT-TB(-)	Sensitivity	Specificity	PPV	NPV	PLR	NLR
	n	n	n	%[95%CI]	%[95%CI]	%[95%CI]	%[95%CI]	%[95%CI]	%[95%CI]
Active TB	68	48	20	70.6[58.9–80.1]		55.8[45.3–65.8]	91.2[86.7–94.2]	4.53[4.23–4.85]	0.35[0.32–0.38]
Culture or histology confirmed TB	12	11	1	91.7[64.6–98.5]		22.5[13.0–35.9]	99.5[97.3–99.9]	5.89[5.50–6.30]	0.10[0.01–0.70]
Clinical diagnosed TB	56	37	19	66.1[52.1–77.8]		49.3[38.3–60.4]	91.6[87.2–94.5]	4.24[3.92–4.59]	0.40[0.36–0.45]
Pulmonary TB	14	13	1	92.9[64.2–99.6]		25.5[15.6–38.9]	99.5[97.3–99.9]	5.96[5.60–6.35]	0.08[0.01–0.60]
Extrapulmonary TB	54	35	19	64.8[50.6–77.0]		48.0[36.9–59.2]	91.6[87.2–94.5]	4.16[3.83–4.52]	0.42[0.38–0.46]
CNS TB	1	1	0	/					
Lymph node TB	2	2	0	/					
Bone and joint TB	4	2	2	/					
Tuberculous serositis	8	6	2	/					
Disseminated TB	2	2	0	/					
Others^a^	3	2	1	/					
No definite site	34	20	14	/					
Active TB excluded	244	38	206		84.4[79.4–88.4]				
CTD	82	11	71		86.6[77.6–92.3]				
Infection	90	17	73		81.1[71.8–87.9]				
Neoplasm	31	5	26		83.9[67.4–92.9]				
Others	41	5	36		87.8[74.5–94.7]				

^a^Others: 2 cases with T.SPOT-TB positive were intestinal TB and pelvic TB respectively; 1 case with T.SPOT-TB negative was adrenal TB.

PPV: positive predictive value; NPV: negative predictive value; PLR: positive likelihood ratio; NLR: negative likelihood ratio.

For 68 patients with ATB and 244 patients with ATB excluded, the numbers of antigen-specific IFN-γ secreting T cells were 314 SFCs/10^6^ PBMC (interquartile range 102–1009) and 96 SFCs/10^6^ PBMC (interquartile range 43–272) respectively (P<0.001), the frequencies of ESAT-6- and CFP-10-specific IFN-γ secreting T cells in patients with ATB were significantly higher than those in patients with ATB excluded (P<0.001). For patients with culture or histology confirmed TB and patients with clinical diagnosed TB, there was significant difference in the numbers of antigen-specific IFN-γ secreting T cells (P<0.01), but there was no significant difference in the frequencies of ESAT-6- or CFP-10-specific IFN-γ secreting T cells. For patients with pulmonary TB and extra pulmonary TB, there were significant difference in the numbers of antigen-specific IFN-γ secreting T cells and in the frequencies of ESAT-6- or CFP-10-specific IFN-γ secreting T cells (P<0.01) (Table 3).

**Table 3:** Frequencies of MTB-specific IFN-γ secreting T cells in patients with and without active TB.

	Frequencies of MTB-specific IFN-γ secreting T cells(SFC/10^6^PBMC) Median[IQR]					
	T-SPOT.*TB*	P	ESAT-6	P	CFP-10	P
Active TB(n = 68)	314[102–1009]	<0.001	140[44–360]	<0.001	372[102–927]	<0.001
Active TB excluded(n = 244)	96[43–272]		66[36–165]		76[52–140]	
Culture or histology confirmed TB(n = 12)	528[120–1036]	0.006	160[100–360]	0.201	396[120–958]	0.056
Clinical diagnosed TB(n = 56)	276[86–1092]		112[37–355]		372[96–938]	
Pulmonary TB(n = 14)	556[344–2302]	0.005	340[78–1026]	0.004	516[212–1116]	0.001
Extrapulmonary TB(n = 54)	208[76–684]		98[43–253]		250[82–868]	

A number of risk characteristics of patients associated with false positive and false-negative results were evaluated in multivariate logistic regression models. ‘Evidence of prior TB’ turned out to be an independent risk factor for false-positive outcomes. Odds ratio (OR) between false-positives and true-negatives was 36.73 (95%CI 11.28–119.61; P<0.001). No risk factors to false-negatives were dentified.

## Discussion

FUO can be a perplexing clinical puzzle. Some studies report as many as 200 different causes of FUO [8]. China is one of the 22 countries recognized by the World Health Organization as having a high TB burden, and the nation has the world’s second largest population of people with this disease. According to the findings of the fifth nationwide tuberculosis epidemiological sampling survey conducted in China in 2010, the prevalence of pulmonary TB remained stable [9]. Many reports reveal that TB, especially extra pulmonary TB, plays an important role in the etiology of FUO in China [4–6]. According to previous case series of FUO in Peking Union Medical College Hospital, ATB was diagnosed in 217 (21.8%) patients among 997 cases with FUO, most of them (186/217, 85.7%) were extrapulmonary TB cases [4].

Many studies already demonstrate the diagnostic value of MTB-specific IGRA in patients with ATB with all clinical types, pulmonary TB, extrapulmonary TB, tuberculous serositis, tuberculous meningitis, and so on. Epidemiological surveys of latent tuberculosis infection (LTBI) showed that age-standardized and sex-standardized positivity rates by an interferon-γ release assay (QuantiFERON [QFT]) ranged from 13% to 20% in China [10]. The high infection rate of LTBI resulted in the lower specificity of T-SPOT.*TB* on PBMC in diagnosis of ATB. Our previous study showed that sensitivity and specificity of T-SPOT.*TB* for diagnosis of ATB in China was 83% and 65% respectively, further analysis showed the sensitivity of T-SPOT.*TB* was 95.7% in patients with culture or histology confirmed TB. In our study, we found the sensitivity and specificity of T-SPOT.*TB* for the diagnosis of ATB in classic FUO patients were only 70.6% (95%CI 58.9–80.1%) and 84.4% (95%CI 79.4–88.4%) respectively, maybe due to most cases were clinically diagnosed TB. Several studies have evaluated the diagnostic value of T-SPOT.*TB* in patients with extrapulmonary TB. Feng et al reported the sensitivity and specificity of T-SPOT.*TB* were 95.6% and 84.1% [11]; Cho et al reported the sensitivity and specificity of T-SPOT.*TB* were 84% and 51% [12]. The diagnostic value of T-SPOT.*TB* varied according to different sites of extrapulmonary TB, it was more sensitive in patients with osteoarticular TB, lymph node TB, genitourinary TB, etc. FUO patients diagnosed as ATB eventually usually presented with atypical manifestations, in our study most extrapulmonary TB patients were ATB with no definite site (34/54) or clinically diagnosed TB (48/54), this maybe the reason why the diagnostic performance of T-SPOT.TB for extrapulmonary TB was moderately below other studies.

While seeking the etiological cause of patients with classic FUO in an high endemic area, ATB is always considered in differential diagnosis. Exclusion of ATB is required for the diagnosis of other diseases such as adult onset Still’s disease, polymyalgia rheumatica, sarcoidosis, Crohn’s disease and so on. Especially when physicians decide to prescribe steroids or immunosuppressants for some patients and observe the clinical effects, it is important to exclude ATB first. Diagnostic anti-TB therapy is a good choice, but it need two to three months, and physicians need to consider the adverse effects of these anti-TB medications. Thus, rapid and accurate exclusion of ATB will be helpful for etiological diagnosis of FUO and thus shorten the duration of diagnosis. IGRAs have been reported to be useful adjunct tests to exclude ATB with all clinical types, pulmonary TB [11], extrapulmonary TB [11–13], intestinal tuberculosis [14], etc. We found the negative predictive value of T-SPOT.*TB* was as high as 91.2% in classic FUO patients, so it may be helpful for excluding ATB in this special population.

IGRA is still difficult to differentiate ATB from LTBI by quantitative scoring of IGRAs [15–17]. A new technique, fluorescence-immunospot which detects dual IFN-γ/IL-2 secreting and single IFN-γ or IL-2 secreting MTB antigen-specific T-cells will be helpful to differentiate ATB from LTBI [18]. We will conduct further study to evaluate the utility of this technique in FUO patients.

The present study has two major limitations. First, many patients with ATB were diagnosed according to clinical criterion rather than culture or histology-confirmed. So, it may under-estimate the diagnostic efficiency of T-SPOT.*TB* assay. Secondly, we enrolled 387 cases with classic FUO, only 68 cases were diagnosed with ATB, it may need more sample to evaluate the diagnostic value of T-SPOT.*TB* in patients with classic FUO.

In summary, TB continues to be an important consideration in the evaluation of patients with classic FUO in China. The high prevalence of prior TB and latent TB infection has an impact on the use of T-SPOT.*TB* for indirect diagnosis. However, T-SPOT.TB appears valuable for excluding active TB among patients presenting with classic FUO, with a high negative predictive value.

## Supporting Information

S1 Table Characteristics, diagnosis and T-SPOT.*TB* of 387 patients with classic FUO. (XLSX)
